# An assessment of Polish women’s level of knowledge about endometriosis: a pilot study

**DOI:** 10.1186/s12905-021-01556-2

**Published:** 2021-12-07

**Authors:** Joanna Szymańska, Magdalena Dąbrowska-Galas

**Affiliations:** 1 Chair of Health Sciences, Faculty of Applied Sciences, WSB University, Dąbrowa Górnicza, Poland; 2 Chair of Physiotherapy, Department of Kinesitherapy and Special Methods, School of Health Sciences in Katowice, Medical University of Silesia, Katowice, Poland

**Keywords:** Endometriosis, Menstruation, Knowledge level

## Abstract

**Introduction:**

Insufficient knowledge about endometriosis among women is one of the causes of its delayed diagnoses. Due to the elusiveness of symptoms, the most important component of early detection is proper and exhaustive knowledge. The objective of the study was to assess Polish women's awareness of endometriosis.

**Methods:**

The pilot studies were performed with the participation of 200 women, in an average age of 33.65 years (*SD* = 11.45), who completed the authors’ questionnaire related to self-assessment of the level of knowledge about the disease, its symptoms, late effects and directions of a remediation procedure. The statistical analysis was performed using the IBM SPSS Statistics 25 suite. It included a frequency analysis, a one-way analysis of variance ANOVA, a single variable regression analysis and Student’s *t*-test for independent samples.

**Results:**

Almost 84% of women had heard about endometriosis, while only 1/3 of them considered their knowledge sufficient or good. Very good knowledge was declared by 4.5% of women, while 16.1% of participants had never heard about it. The level of knowledge was significantly higher (*p* = 0.001) among women with medical education. Polish women acquired their knowledge mainly from the Internet and the experience of other women. The reasons identified by women were the still existing ‘taboo’ related to menstruation, the absence of information in the media and education in schools, which is critical according to 92.4% of women.

**Conclusions:**

Polish women’s level of knowledge about endometriosis is insufficient, which we should strive to improve. Higher awareness is presented by women with medical education, and the higher the level of knowledge, the larger a woman’s interest in healthy behaviour.

## Introduction

Endometriosis is a chronic, estrogen-related inflammatory disease, and it is characterized by the presence of endometrial-like tissue beyond the uterine cavity. Severity and clinical presentation are very varied of this systemic disease that predominantly affecting the pelvis [1, 2]. The most common sites of endometriosis are the ovaries, fallopian tubes, Douglas sinus peritoneum, pelvis and abdominal peritoneum. In the case of deeply infiltrating endometriosis, disease foci also occur in the bladder and intestines [3].

Endometriosis is an exceptionally heterogeneous disease, which can cause minor symptoms among some women, and an agonising pain among others, affecting their professional life, social contacts or reproduction [4–6]. Also, the growing intensity of symptoms is often not correlated with the severity of the disease [5–8]. One of its most troublesome symptoms is the overwhelming pain, which can occur as nonmenstrual pain in the lesser pelvis (10–50%), cyclic pain during menstruation (28–80%), pain during sexual intercourse in women with deeply infiltrating endometriosis, disrupting sexual life (12–40%) or defecation [1, 6–11]. Over time, the disease leads to infertility, which affects 30–50% of women [2, 4, 7, 12]. These symptoms affect the physical and emotional well-being of women, seriously disrupting the quality of their lives [13–15]. Laparoscopic eradication of endometriosis includes patients who did not respond to hormonal therapy and those who present proper surgical indication (i.e., hydroureteronephrosis, subocclusive bowel involvement) [16]. Multivisceral surgery is often mandatory in deeply intiltrating endometriosis with a tailored and modular approach, which can lead in some cases to segmental bowel resection and/or ureterovesical reimplantation [17].

It is assumed that endometriosis affects approximately 10–15% of the population of women of childbearing age, and 2–5% women after menopause, which amounts to 176 million of women in the whole world [7, 9, 11]. Although rare in postmenopausal women, endometriosis should be considered in the differential diagnosis of pelvic diseases. Sometimes abdominal pain, due to retroperitoneal endometriosis, may cause deep vein thrombosis [18].

Most of incidence data comes from American, British, Canadian and Australian epidemiological research [1, 5, 8, 10, 15, 19–21]. So far, wide spread research has not been performed in Poland. In the Ministry of Health’s statistics for 2018, there are only 14,000 women with endometriosis who have been treated in a hospital (3560 women) or an outpatient clinic (10,461 women) [22]. However, the unofficial number in Poland may be as high as 2 million women [23].

Due to the heterogeneity of symptoms, endometriosis is diagnosed with much delay. In the world, the average time of waiting for a proper diagnosis is currently 8–10 years, depending on the country [4, 8, 10, 14, 21, 24, 25]. Reasons for this are sought in ignoring its early symptoms, especially those related to menstrual pain [4, 8–11]. For centuries, the menstrual pain was a taboo, an embarrassing problem and an element of a woman’s life. There is still a popular opinion that it is a kind of women’s ‘thing’ which does not require the implementation of diagnostics [19, 22], and women who complain are perceived as exaggerating the symptoms, or suffering from diseases of a psychosomatic nature [26–29]. On the other hand, in accordance with the recommendations of the European Society of Human Reproduction and Embryology (ESHRE), in each case of the occurrence of ailments related to the menstrual cycle, after the exclusion of their other causes, endometriosis is to be suspected [2, 30].

Endometriosis is also a costly problem of public health. The average annual cost of treatment per one patient was calculated in 10 countries at € 9,579. These costs result mainly from surgeries, monitoring examinations, hospitalisation, as well as medical visits, and they grow along with the increasing intensity of endometriosis [1, 8, 13, 24, 31].

All over the world, actions are taken in order to build social awareness of endometriosis by various social campaigns and actions of the associations of sick women [25]. Since 2014, worldwide EndoMarches have been organised in March, the month of building this awareness, uniting over 60 countries in the world under one common objective: to eliminate the taboo and replace it with dialogue [32]. In 2019, for the first time Polish women also joined the rest of the world, believing that this march can be a good beginning of a change in the approach to women with endometriosis, including in Poland [23].

Due to the scale of the problem, it is becoming a priority to increase social awareness about endometriosis. In the literature, there is available data related to the awareness of other diseases, but there are few papers related to the knowledge about endometriosis in the world, with the total absence of them in relation to Poland [33–35]. Seeing an enormous need for changes in this regard, the authors of the present paper committed themselves to assessing women’s level of knowledge about endometriosis.

## Method

The pilot studies were performed with the participation of 232 women who were more than 18 years of age. The final analysis took into account 200 individuals; 32 surveys were incomplete. Due to the lack of a standardised questionnaire, the research tool consisted of the authors’ own survey, created based on the review of the related literature [36].

The research was performed upon an approval of the ethical commission for scientific research of the WSB University in Dąbrowa Górnicza, No. AWSB/KE/02/2020. Informed consent was obtained from all participants.

The questionnaire was preliminarily tested among 10 people and subsequently, after making the necessary corrections, formatted for the needs of an internet poll and uploaded to the generally available Google Forms Platform, where it was accessible for completion from 10 June 2020 till 30 September 2020. In order to acquire a sample of the target population, the recruitment of participants proceeded by publishing a link to the questionnaire in a social media platform (Facebook). Before collecting the data of the respondents, they were informed about the voluntariness and anonymity of participation in the research. It took about 15–20 min to complete the Internet poll.

The survey was divided into two parts. The first one involved sociodemographic variables of the participants (age, sex, level and direction of education, occupation and professional status, place of residence). In the second part of the poll, there were 45 questions which were closed-ended in nature, and providing a possibility to choose one or several answers among the listed options. The questions referred to self-assessment of the level of knowledge about endometriosis, as well as the sources of this knowledge, reasons behind possible lack of knowledge, the age of women who are mainly affected by this disease, the time necessary to make a proper diagnosis, first atypical symptoms and effects of the disease, as well as a need for education in that regard.

The criterion of inclusion into the research consisted of a female gender, an age above 18 years and completing the questionnaire in its entirety, while a male gender, an age below 18 years or an incomplete questionnaire meant exclusion from the research. All methods were carried out in accordance with relevant guidelines and regulations.

In order to provide answers to the presented research questions, statistical analyses were performed using the IBM SPSS Statistics 25 suite. It enabled the performance of a frequency analysis, a one-way analysis of variance ANOVA, a single variable regression analysis and Student’s *t*-test for independent samples. In order to determine the relations, an analysis was performed based on cross tabulation and a chi-squared test of independence. The significance level adopted in the present chapter was α = 0.05.

## Results

The highest number of respondents had higher education (48.5%) and lived in urban areas (79%). 76% of women were professionally active; 43.5% of respondents pursued a medical profession (Table 1).

**Table 1:** Sociodemographic characteristics and the level of knowledge about endometriosis of the surveyed women (n = 200)

Variables	n	%
*Education level*		
Full secondary	48	24
Incomplete higher (bachelor’s degree)	31	15.5
Higher	97	48.5
None—student	26	13
*Education*		
Medical	87	43.5
Nonmedical	113	56.5
*Professional status*		
Student/pupil	26	13
Professionally active	152	76
Unemployed	19	9.5
Retired	3	1.5
*Place of residence*		
Town	158	79
Suburban areas	10	5
Village	32	16
*Degree of knowledge about endometriosis*		
Very good	9	4.5
Good	55	27.5
Sufficient	61	30.5
Satisfactory	18	9
Insufficient	26	13
Very bad	31	1.5
Sources of knowledge*		
Internet	90	45
Experience of other women	72	36.1
Physician	33	16.3
Own experience	31	15.3
Reasons behind the lack of knowledge*		
Insufficient information in the media	128	64
Belief that menstrual pain is a standard attributed to gender, not a symptom	107	53.5
A ‘taboo’ related to menstruation	132	66
No information about the disease from physicians	62	31.4
No education in schools about menstrual pain and women’s diseases	76	38
Aspect of the disease, to which the lack of knowledge was related*		
Diagnosing time	166	83.2
The occurrence of infertility as a consequence of the untreated disease	145	72.5
Menstrual pain as the earliest symptom of the developing disease	127	63.3
The age of women who most frequently suffer from endometriosis	43	21.3

*The number n does not equal 200, due to the possibility of giving more than one answer

An analysis of the frequency of answers to questions from the poll related to the knowledge of women about endometriosis indicated that most of the surveyed women (83.9%) knew what endometriosis is, while in terms of self-assessment of the level of their knowledge, the most women stated that it was sufficient or good. On the other hand, the lowest number declared very good or satisfactory knowledge. In turn, 16.1% of respondents had never heard about the disease.

Most women acquired their knowledge about the disease from the Internet (45%), less from the experience of other women (36.1%), and the lowest number got it from their own experience (15.3%). The women see the reasons behind the lack of knowledge primarily in an insufficient amount of information on this subject in the media (64%), the belief that menstrual pain is a standard attributed to the gender and not a disease symptom (53.5%), and the widespread ‘taboo’ related to menstruation (66%) (Table 2).

**Table 2:** Knowledge about endometriosis depending on medical and nonmedical education

*M*	Medical (*n* = 87)		Nonmedical (*n* = 113)		95% *CI*				
		*SD*	*M*	*SD*	*t*	*p*	*LL*	*UL*	*Cohen's d*
Knowledge about endometriosis	3.63	1.31	2.85	1.40	4.02	0.001	0.40	1.17	0.58

Women’s lack of knowledge involved the fact that diagnosing the disease requires a period of many years (83.2%), the possibility of the occurrence of infertility as a consequence of the untreated disease (72.5%), or finally that the disease primarily affects women in the period of full reproductive maturity (21.3%) (Table 1).

Among women with medical education, knowledge about the disease was statistically significantly higher (*p* = 0.001) than among women lacking such education (Table 2). On the other hand, neither the age nor the place of residence were predictors of knowledge about endometriosis—this relationship turned out to be statistically insignificant (Table 3).

**Table 3:** Age as the predictor of knowledge about endometriosis

	*B*	*SE*	*Beta*	*t*	*R*^2^	*F*
(Constant)	3.17	0.31		10.17	0.001***	0.01**
Age	0.001***	0.01**	0.001***	0.03*		

Place of residence as the predictor of knowledge about endometriosis									
	Town (*n* = 158)		Suburban areas (*n* = 10)		Village (*n* = 29)		*F*	*p*	η^2^
	*M*	*SD*	*M*	*SD*	*M*	*SD*			
Knowledge about endometriosis	3.22	1.35	2.50	1.78	3.21	1.54	1.23	0.295	0.01

*—*p* < 0.05; **—*p* < 0.01; ***—*p* < 0.001

The approach of the surveyed women to menstrual pain was also analysed. It turned out that 85% of them believe it to be an abnormal symptom, while the remaining consider it a standard. An analysis of relations was performed for women who admitted that they experience strong menstrual pains, and had a varying approach to the normality of their occurrence. However, it turned out to be statistically insignificant (Table 4). 66.5% of women also admitted that menstruation is still a ‘taboo’ in Poland. Therefore, the relationship between the approach to menstrual pain and the consideration of menstruation as a taboo was also analysed. In this regard, the analysis did not exhibit statistical significance either (Table 4).

**Table 4:** Relationships between the occurrence of menstrual pain and normality of the course of menstruation/a taboo subject

The occurrence of painful menstruation	Continuous menstrual pains are—normal		Continuous menstrual pains are—abnormal		Total	
	*N*	%	*N*	%	*N*	%
*Relationship between the occurrence of painful menstruation and consideration of this pain as a normal process*						
Yes	8	26.7	57	33.5	65	32.5
No	22	73.3	113	66.5	135	67.5
Total	30	100	170	100	200	100
χ^2^(1) = 0.55; *p* = 0.303						

Menstruation is still a ‘taboo’ in Poland	Continuous menstrual pains—normal		Continuous menstrual pains—abnormal		Total	
	*N*	%	*N*	%	*N*	%
*Relationship between considering menstrual pains normal or not and menstruation itself as a taboo*						
Yes	21	70	112	65.9	133	66.5
No	9	30	58	34.1	67	33.5
Total	30	100	170	100	200	100
χ^2^(1) = 0.19; *p* = 0.415						

32.5% of the surveyed women experiencing strong menstrual pains admitted that they had accepted and ignored them so far, mainly due to shame, embarrassment and fear of ridicule during possible reporting of such concerns to a physician (Table 5). As many as 90.5% claimed that such an approach towards menstruation should change. 92.4% of women also think that education about endometriosis is necessary, and that it could result in a number of benefits (Table 5). They also believe that, since menstrual pains experienced already by young girls are the first symptom which can indicate the presence of the disease, then education in that regard should be primarily handled by schools, followed by the media and physicians (Table 5).

**Table 5:** Reasons behind the acceptance of menstrual pain, benefits from education and its indicated sources

Reasons behind the acceptance and ignorance of menstrual pain*	%
Shame	53.3
Embarrassment	49.2
Fear of ridicule	68.3
Benefits resulting from education about endometriosis*	
More careful observation of signals sent by one’s own body	83.7
Consultation of one’s concerning symptoms with a physician	68.8
Prevention of the deteriorating quality of life for women suffering from the pain	60.4
Decreasing the percentage of women with infertility	54.5
Increasing the awareness that it is a disease, not an inherent feature of women	23.2
Indicated sources of education*	
School	83.2
Media	63.9
Physicians	53.5

*The % value does not equal 100, due to the possibility of giving more than one answer

## Discussion

Endometriosis is a disease with an increasing frequency of occurrence in the populations of all countries in the world. Its early diagnosis gives women a chance to return to normal everyday functioning without pain. The lack of knowledge about its symptoms and effects, and thus their frequent ignorance, delays visits to medical specialists, and therefore prolongs a proper diagnosis and the initiation of treatment.

The authors’ own research results indicated that, although most women declared that they had heard about the existence of endometriosis, their knowledge in this regard is definitely inefficient, and 16.1% of them lack it entirely. In the research of Moradi et al., covering 35 women suffering from endometriosis, many of them had never heard about this disease before being diagnosed either [24]. On the other hand, 52% of those surveyed by Shadbolt et al. had heard about the existence of endometriosis [36]. Similar observations were made by Bush et al. while performing research in New Zealand in 2015, where 32% of students from Canterbury and 18% in Hawkes Bay were aware of its existence [31].

In our research, a higher level of knowledge was demonstrated by women with medical education, while neither the age nor the place of residence were predictors of knowledge about endometriosis. Among women who claimed that they had heard about the disease, most of them had learned about it from the Internet, or from other women suffering from it. Only some of them acquired this knowledge from a physician. Similar answers were provided by women surveyed in the paper of Facchin et al., who, due to unpleasant contacts and relations with physicians, would rather use the Internet to acquire information about the disease [26].

Women with endometriosis, especially with pain symptoms present during menstruation, face a social stigma and a wide range of various reactions of physicians when looking for medical help in that regard. Consideration of the symptoms of endometriosis as ‘a part of the normal life of women’ seems to be an experience shared by women with endometriosis all over the world. This is mentioned in the papers of Grundström [4], Moradi et al. [24], Culley et al. [21], as well as Riazi et al. [37]. Facchin et al. [26] pointed out that women reporting to physicians due to menstrual pain were facing the lack of knowledge, empathy or support, and they were treated by the medical personnel with ignorance and incredulity. The women felt embarrassed when they were describing to the physicians how the pain and bleeding were limiting and controlling their everyday lives. In the authors’ own research, 32.5% of women experiencing their own strong menstrual pains also ignored the problem, mainly due to fear of ridicule, shame and embarrassment.

Research by Arena et al. shows that women with endometriosis also show a high level of anxiety at the diagnostic stage, especially if their knowledge about the disease is insufficient. The authors assessed the level of anxiety in 104 women with suspected endometriosis and observed that those women who did not know the disease and those who searched for information on the Internet, had a higher baseline anxiety level compared to women whose level of knowledge about the disease was higher. These women also had greater reduction in post-visit anxiety. The authors believe that building a trustful relationship between a doctor and women, as well as providing them with tailored counseling are essential to improve their psychological wellbeing [38].

Infertility turned out to be the most concerning consequence of the untreated disease both among those women who declared at least sufficient knowledge about the disease, as well as those who lacked this knowledge. Navarria-Forney et al. also showed that most women (96%) were worried about the impact of endometriosis on fertility. Importantly, most of them considered themselves insufficiently informed about the possibilities of maintaining fertility [39].

Almost all of the surveyed women (92.4%) were also interested in extending their knowledge about the disease. They indicated that education should be handled primarily by schools, followed by the media and physicians. In the papers of Shadbolt et al., the necessity of education, especially among teenagers, is expressed by as many as 89% of the surveyed women, while any health-promoting actions should be directed towards sources preferred especially by young women: schools (40%), the Internet (22%) and magazines (13%). Through education, the participants in the studies would like to learn about the symptoms, risk factors and treatment of the disease, similarly to the respondents from our research [32]. The key significance of education on menstrual pain experienced by teenage women was also described by DiVasta et al., who claimed that the promotion of proper behaviour could be related to care and a decrease in the delays of endometriosis diagnostics [11].

There is also strong suggestive evidence that a consistently managed programme of education on menstrual health in schools increases the awareness of teenage girls about endometriosis. This was proven by researchers from New Zealand, who ran an audit of education programmes related to menstrual health and endometriosis introduced in secondary schools, and conducted observations regarding the number of young women reporting to physicians for this reason. It turned out that in the region in which the educational programme was consistently pursued, the awareness of girls regarding endometriosis was 32%. Moreover, a shift was also observed in terms of young women’s earlier contacts with specialised health care [30]. A similar opinion was expressed by respondents in the papers of Hudelist et al. [25] and Moradi et al. [24].

An outline of a remediation programme should be created based on the yielded research results, the priority of which will be to decrease the number of cases of endometriosis in Poland, or at least its effective diagnosing and treatment.

## Conclusions

Polish women's level of knowledge about endometriosis is insufficient, but higher among women with medical education. Furthermore, it has been concluded that the correlation between higher levels of education is interconnected with a woman’s interest in healthy behaviours. The indication of symptoms and effects of the untreated disease turned out to be a sufficient reason to convince women to consult a physician. The research will continue with a larger target group, since there is a need for wide investigation of the subject and education in that regard.

## Data Availability

The datasets used and/or analysed during the current study are available from the corresponding author on reasonable request.
